# Comparative Efficacies of Linezolid vs. Tedizolid in an Experimental Murine Model of Vancomycin-Resistant Enterococcal (VRE) Bacteremia

**DOI:** 10.3389/fmed.2019.00031

**Published:** 2019-02-20

**Authors:** Wessam Abdelhady, Nagendra N. Mishra

**Affiliations:** ^1^Division of Infectious Diseases, Los Angeles Biomedical Research Institute at UCLA Medical Center, Torrance, CA, United States; ^2^David Geffen School of Medicine at UCLA, Los Angeles, CA, United States

**Keywords:** TZD, LZD, murine model, VRE, bacteremia

## Abstract

Tedizolid (**TZD**) is an oxazolidinone derivative which demonstrates bacteriostatic activity through inhibition of protein synthesis. We compared the efficacies of TZD and an earlier-generation oxazolidinone, linezolid (**LZD**), in an experimental murine model of bacteremia caused by two VRE strains (one each *E. faecium* and *E. faecalis*). LZD exhibited significantly better efficacy in terms of reduced VRE blood and target tissue densities than TZD in this model.

## Text

Infections caused by enterococci (especially VRE strains) are major problems worldwide (1–4). VRE strains, particularly in immunocompromised patients, are a major and steadily increasing cause of bacteremia (5). VRE bloodstream infections (**VRE-BSI**) rank as one of the top four major infections among hospitalized patients (5). Commonly used antimicrobial agents used to treat VRE-BSI infections, such as daptomycin, oritavancin, dalbavancin, quinupristin-dalfopristin, and tigecycline, each have a number of important side effects, both acutely and more long-term (2). Currently, optimal treatment for VRE-BSI remains problematic and undefined. Thus, plausible alternative approaches for the treatment of such infections are urgently required.

We have recently employed a discriminative murine model of acute VRE-BSI to measure the host's ability to control infection and the impact of selected therapeutic interventions (1, 2). TZD is an oxazolidinone-class antibiotic, approved by the FDA in 2014, for the treatment of acute bacterial skin and skin structure infections (ABSSSI) caused by certain bacteria, including MRSA,VRE and various *Streptococcus* species (3). Recently Sahm et al. evaluated TZD's overall activity and emergence of resistance in gram-positive bacteria (3). In addition, the comparative efficacy of LZD and daptomycin in the same experimental murine model of VRE bacteremia were reported before (1, 2). However, to our knowledge, there have been no studies that have examined a head-to-head comparative efficacy analysis of TZD vs. LZD in this experimental VRE-BSI model.

In parallel to *in vivo* studies, we also tested TZD vs. LZD to assess their comparative capacities to exert *in vitro* bactericidal impacts on both VRE study strains. The LZD and TZD MICs (minimum inhibitory concentration; lowest concentration of antibiotics which kills or prevents the minimum growth of the bacteria) of the 613 and 447 VRE strains were carried out by microbroth dilution assay (6). We then employed a range of LZD and TDZ concentrations in time-kill curves, ranging from sub-MIC to 5 × MIC, utilizing an initial inoculum of ~1 × 10^5^ CFU/mL (7). We selected the following incubation time-points, for analyses (0, 2, 4, 6, 24 h of incubation at 37°C). Thus, an aliquot of all reaction tubes was quantitatively cultured at each time-point (7). Summary data were expressed as mean log_10_ CFU/mL (±SD) of surviving counts. A minimum of two experimental runs were performed on separate days. A “bactericidal effect” was defined as at least a 3 log_10_ CFU/mL reduction in counts as compared to the initial inoculum (7).

In this investigation, we studied the comparative efficacy of TZD vs. LZD in an acute murine VRE-BSI model, using two well-characterized VRE strains (*E. faecalis* 613 and *E. faecium* 447) (4, 6); For inducing this experimental infection, a tail vein challenge of BALB/c mice with our two VRE study strains above was done. The final inoculum utilized for each strain (1 × 10^9^ CFU/mL) was determined by a number of pilot studies that showed significant infection levels in the blood and three target organs (lung, kidney, spleen) in each animal, without excessive mortality (see below). The mice were purchased from Jackson Lab Laboratory, Bar Harbor ME. All studies were approved by the Los Angeles Biomedical Research Institute IACUC Committee.

In brief, our initial pilot studies were focused on determining the inoculum of the two VRE strains that resulted in a at least a 95% infectious dose (ID_95_) for this murine model. The ID_95_ for this study represented the optimal inoculum resulting in a non-lethal, reliable and durable infection for 95% of animals. For these inoculum-ranging studies, infection was given by tail vein challenge of BALB/c mice at a 10^6^, 10^7^, 10^8^, or 10^9^ CFU/mL inocula. These inocula represent a standard inoculum range for this murine model of VRE-BSI. At 24 h post-challenge, animals were sacrificed, and blood, kidney spleen and lung were sterilely removed and quantitatively cultured in BHI media (1, 2). Individual target organ summary data were expressed as either log_10_ CFU/mL blood or log_10_ CFU/g tissue.

On the basis of the ID_95_ data, (see Supplementary Table 1), BALB/c mice were given a tail vein challenge of the 1 × 10^9^ CFU/mL inoculum, then randomized at 24 h post-infection to receive either: (i) no therapy (controls); (ii) LZD for 3 d; or (iii) TZD for 3 d. The dose-regimen of LZD and TZD were: 120 mg/kg SC bid, or 10 mg/kg/day ip, respectively. These doses were selected for the *in vivo* studies based on their simulated targeted human PK-PD profiles (8, 9). At least 24 h after the last TZD or LZD dose (day 4), one-half of surviving mice were euthanized for an end-of-therapy evaluation (**EOT**), with blood, kidney, spleen and lung removed and quantitatively cultured as above. For the remaining one-half of surviving animals, an evaluation of “relapse” was carried out on day 8 (i.e., after 4 antibiotic treatment-free days); at sacrifice, the blood and target tissues were again removed and quantitatively cultured as above (1, 2).

The two-tailed Student *t*-test was used for statistical analyses of quantitative data. *P* ≤ 0.05 were considered significant.

**Figure 1:**
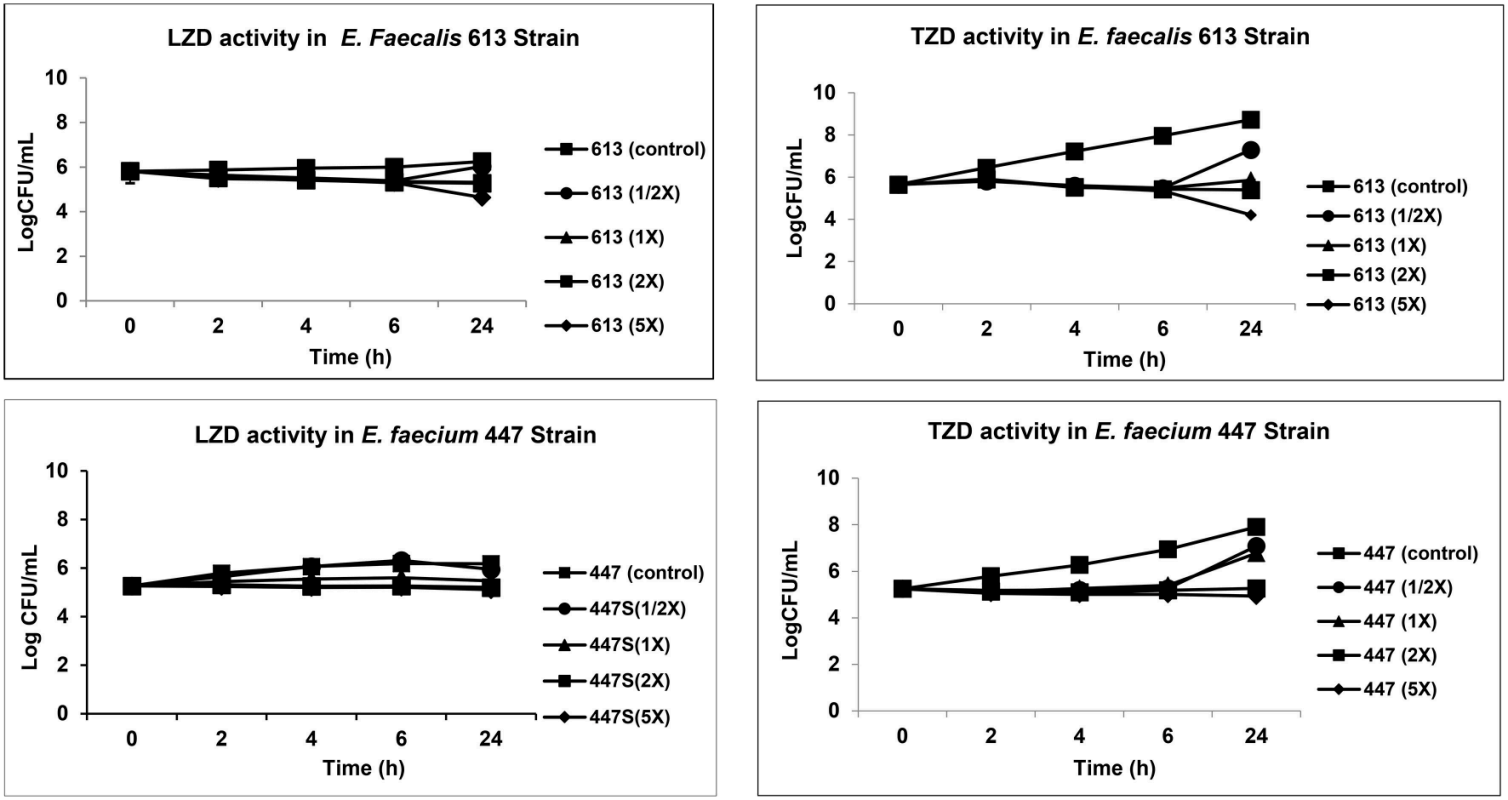
Comparative analysis of TZD and LZD activity by *in vitro* time-kill curve assay.

The mean TZD and LZD MICs were (0.25 and 0.5 μg/mL) and (1.0 and 2.0 μg/mL) for 447 and 613 VRE strains, respectively. As shown in Figure 1, (Supplementary Table 2), neither LZD nor TZD was bactericidal for either VRE strain. Thus, both agents were essentially bacteriostatic against the VRE study strains *in vitro*.

In the VR-*E. faecium* 447 strain, in comparing the treatment and relapse outcomes of LZD vs. TZD, LZD resulted in a significantly lower kidney bacterial burden (*P* < 0.05); this same difference was not found in other target tissues (Table 1). Similarly, LZD significantly prevented the relapse of infection with this strain in the kidney. Further, the treatment outcome of LZD vs. TZD in VR-*E. faecalis* 613 strain, LZD treatment yielded significantly lower CFU counts in all target organs (*P* < 0.05) (Table 1). However, the relapse group microbiologic outcomes comparing LZD vs. TZD for this strain were similar, and the data were not statistically significant (Table 1).

**Table 1:** VRE-BSI using 447 *E. faecium* and 613 *E. faecalis* comparing LZD and TZD in terms of tissue burden (CFUcount) [log CFU/g ±SD] at EOT on day 4.

**Strains**		**Antibiotics**	**Kidney**	**Spleen**	**Lung**	**Blood**
447S (no. of mice)	Treatment (7)	TZD	6.06 ± 0.64	2.58 ± 0.60	2.93 ± 0.66	0
		LZD	5.43 ± 0.25^*^	2.97 ± 0.25	3.13 ± 0.47	0
	Relapse (7)	TZD	7.07 ± 1.11	3.05 ± 0.99	3.73 ± 0.95	1.28 ± 1.19
		LZD	5.28 ± 0.46^*^	3.15 ± 0.84	3.44 ± 0.64	0.94 ± 0.69
613S (no. of mice)	Treatment (7)	TZD	7.82 ± 0.38	4.72 ± 0.81	5.39 ± 0.36	2.11 ± 0.92
		LZD	5.97 ± 1.05^*^	4.00 ± 0.38^*^	3.80 ± 0.31^*^	0.56 ± 0.72^*^
	Relapse (7)	TZD	7.49 ± 0.56	4.33 ± 1.23	4.43 ± 1.14	2.17 ± 0.84
		LZD	7.56 ± 0.90	4.29 ± 0.86	4.29 ± 0.73	2.26 ± 0.93

* *P < 0.05 TZD vs. LZD*.

In our study, neither LZD nor TZD was highly efficacious in this murine VRE-BSI model, although, overall, LZD seemed to yield better treatment outcomes than TZD. Paradoxically, neither drug was able to exert a bactericidal effect *in vitro* against either VRE strain. This suggested that the modestly enhanced *in vivo* impact of LZD in this model may relate to an *in vivo* “synergy” between this agent and innate host defense cells (e.g., PMNs) and/or molecules (e.g., host defense peptides; antibody, complement, etc.) (10, 11). In contrast to our data, there have been several studies carried out by various groups, including us, regarding the comparative activity of TZD vs. LZD in *Staphylococcus aureus*, enterococci and *Streptococcu*s *pneumonia*, employing distinct *in vitro* and *in vivo* scenarios, suggesting that TZD had significantly better activity than LZD (12–16).

We recognize that our current study have methodologic challenges which somewhat limit interpretation. For example, we studied only one *E.faecium* and *E. faecalis* strain in single *in vivo* model. Thus, further studies (including murine BSI model) in the distinct *in vivo* infection model such as endovascular and skin and soft tissue infection model etc., comparing TZD and LZD, and utilizing additional strains of VR-*E. faecium* and VR-*E. faecalis*, will be required to adjudicate our observations.

## Ethics Statement

Animals were maintained in accordance with the American Association for Accreditation of Laboratory Animal Care criteria and were cared for in accordance with national guidance. Animal studies were approved by the LABioMed at Harbor-UCLA Medical Center IACUC Committee.

## Author Contributions

All authors listed have made a substantial, direct and intellectual contribution to the work, and approved it for publication.

### Conflict of Interest Statement

The authors declare that the research was conducted in the absence of any commercial or financial relationships that could be construed as a potential conflict of interest.
