# Supplementary material for: Comparative Efficacies of Linezolid vs. Tedizolid in an Experimental Murine Model of Vancomycin-Resistant Enterococcal (VRE) Bacteremia
Source: Front Med (Lausanne). 2019 Feb 20;6:31. doi: 10.3389/fmed.2019.00031 (PMC6391330; doi:10.3389/fmed.2019.00031)
Supplement: Supplementary file 1 [file Data_Sheet_1.docx]

**Supplementary Table-1. ID_95_** **of 447 *E. faecium* and *613* *E. faecalis* strains**

| **Strains** | **Number of mice** | **Inoculum size** | **Kidney** | **Spleen** | **Lung** | **Blood** |
| --- | --- | --- | --- | --- | --- | --- |
| **447** | **10** | **10^6^** | **2.44±0.54** | **2.72±0.24** | **2.30±0.91** | **0** |
|  |  | **10^7^** | **3.84±0.60** | **3.94±0.26** | **3.19±1.13** | **0.33±0.50** |
|  |  | **10^8^** | **4.72±0.55** | **4.50±0.33** | **3.99±0.39** | **0.95±0.75** |
|  |  | **10^9^** | **8.16±0.23** | **6.86±0.26** | **8.34±0.61** | **4.21±0.31** |
| **613** | **10** | **10^6^** | **4.53±0.81** | **4.36±0.53** | **4.14±0.81** | **1.34±0.38** |
|  |  | **10^7^** | **5.94±0.37** | **5.69±0.25** | **4.65±0.24** | **2.36±0.50** |
|  |  | **10^8^** | **6.22±0.38** | **5.84±0.30** | **5.12±0.16** | **2.11±0.33** |
|  |  | **10^9^** | **6.55±0.43** | **6.53±0.29** | **5.91±0.66** | **2.73±0.45** |

**Supplementary Table-2. Comparative analysis of TZD and LZD activity by *In-vitro* time-kill assay**

**LZD activity in *E. Faecalis* 613 Strain**

| **Time (hr)** | **Control** | **0.5X** | **1X** | **2X** | **5X** |
| --- | --- | --- | --- | --- | --- |
| **0** | **5.8 ± 0.0** | **5.8 ± 0.0** | **5.8 ± 0.0** | **5.8 ± 0.0** | **5.8 ± 0.0** |
| **2** | **5.9 ± 0.1** | **5.6 ± 0.1** | **5.5 ± 0.0** | **5.5 ± 0.0** | **5.5 ± 0.0** |
| **4** | **5.9 ± 0.0** | **5.5 ± 0.1** | **5.5 ± 0.0** | **5.4 ± 0.1** | **5.4 ± 0.1** |
| **6** | **6.0 ± 0.0** | **5.4 ± 0.0** | **5.4 ± 0.0** | **5.3 ± 0.0** | **5.3 ± 0.0** |
| **24** | **6.2 ± 0.0** | **6.0 ± 0.1** | **5.3 ± 0.0** | **5.3 ± 0.1** | **4.6 ± 0.2** |

**LZD activity in *E. Faecium* 447 Strain**

| **Time (hr)** | **Control** | **0.5X** | **1X** | **2X** | **5X** |
| --- | --- | --- | --- | --- | --- |
| **0** | **5.3 ± 0.1** | **5.3 ± 0.1** | **5.3 ± 0.1** | **5.3 ± 0.1** | **5.3 ± 0.1** |
| **2** | **5.8 ± 0.0** | **5.6 ± 0.1** | **5.4 ± 0.0** | **5.3 ± 0.1** | **5.2 ± 0.2** |
| **4** | **6.1 ± 0.2** | **6.1 ± 0.1** | **5.6 ± 0.4** | **5.3 ± 0.1** | **5.2 ± 0.1** |
| **6** | **6.2 ± 0.2** | **6.3 ± 0.2** | **5.7 ± 0.4** | **5.3 ± 0.1** | **5.2 ± 0.1** |
| **24** | **6.2 ± 0.3** | **5.9 ± 0.2** | **6.0 ± 0.2** | **5.2 ± 0.1** | **5.1 ± 0.3** |

**TZD activity in *E. Faecalis* 613 Strain**

| **Time (hr)** | **Control** | **0.5X** | **1X** | **2X** | **5X** |
| --- | --- | --- | --- | --- | --- |
| **0** | **5.7 ± 0.1** | **5.7 ± 0.1** | **5.7 ± 0.1** | **5.7 ± 0.1** | **5.7 ± 0.1** |
| **2** | **6.4 ± 0.0** | **5.9 ± 0.1** | **5.9 ± 0.1** | **5.9 ± 0.0** | **5.8 ± 0.0** |
| **4** | **7.2 ± 0.0** | **5.5 ± 0.0** | **5.5 ± 0.0** | **5.5 ± 0.0** | **5.6 ± 0.0** |
| **6** | **8.0 ± 0.1** | **5.5 ± 0.0** | **5.5 ± 0.0** | **5.4 ± 0.0** | **5.3 ± 0.0** |
| **24** | **8.7 ± 0.1** | **5.9 ± 0.1** | **5.9 ± 0.1** | **5.4 ± 0.0** | **4.2 ± 0.1** |

**TZD activity in *E. Faecium* 447 Strain**

| **Time (hr)** | **Control** | **0.5X** | **1X** | **2X** | **5X** |
| --- | --- | --- | --- | --- | --- |
| **0** | **5.2 ± 0.0** | **5.2 ± 0.0** | **5.2 ± 0.0** | **5.2 ± 0.0** | **5.2 ± 0.0** |
| **2** | **5.8 ± 0.1** | **5.2 ± 0.1** | **5.1 ± 0.0** | **5.1 ± 0.0** | **5.1 ± 0.0** |
| **4** | **6.3 ± 0.0** | **5.2 ± 0.0** | **5.3 ± 0.0** | **5.1 ± 0.0** | **5.0 ± 0.0** |
| **6** | **6.9 ± 0.0** | **5.3 ± 0.0** | **5.4 ± 0.0** | **5.2 ± 0.0** | **5.0 ± 0.1** |
| **24** | **7.9 ± 0.1** | **7.1 ± 0.1** | **6.8 ± 0.0** | **5.3 ± 0.0** | **4.9 ± 0.1** |
